# A Systematic Review of Sodium-Glucose Cotransporter 2 (SGLT2) Inhibitors and Sympathetic Nervous System Inhibition: An Underrated Mechanism of Cardiorenal Protection

**DOI:** 10.7759/cureus.26313

**Published:** 2022-06-25

**Authors:** Shafaat Raza, Stephen Osasan, Sudiksha Sethia, Tayyaba Batool, Zarna Bambhroliya, Joel Sandrugu, Michael Lowe, Oluwasemilore Okunlola, Pousette Hamid

**Affiliations:** 1 Research, California Institute of Behavioral Neurosciences & Psychology, Fairfield, USA; 2 Neurology, California Institute of Behavioral Neurosciences & Psychology, Fairfield, USA

**Keywords:** type 2 diabetes mellitus, blood pressure, drug effects, heart rate, cardiac protection, sympathetic overactivity, autonomic nervous system, sympathetic nervous system, sglt2 inhibitor, sodium-glucose transporter 2 inhibitors

## Abstract

Sodium-glucose cotransporter 2 (SGLT2) inhibitors have many actions beyond glycemic control. The drug leads to favorable cardiovascular and renal outcomes. In this review, we focused on how SGLT2 inhibitors produce these outcomes and what role it plays in the inhibition of the sympathetic nervous system in diabetic patients.

We searched PubMed, Google Scholar, and Biomed Central databases from January 2016 to February 2022. The authors used specific keywords and the Medical Subject Heading (MeSH) strategy. We identified a total of 3,961 records. Strict inclusion-exclusion criteria were followed to gather relevant data. From 3,961 results found through electronic databases, we finally selected 161 studies after the removal of duplicates, excluding irrelevant studies and those that did not fall into inclusion criteria. Forty-one studies underwent an extensive content search and quality appraisal using specific tools. It included a total of 12 best studies to conduct the systematic review supporting data from 17 other studies. Our review found that the SGLT2 inhibitors significantly reduced cardiovascular endpoints, including cardiovascular death, heart failure hospitalization, and all-cause mortality, with varying effects on major adverse cardiovascular (MACE). There were nominal improvements in renal outcomes (decline in renal disease progression, decreased albuminuria, less need for renal replacement therapy [RRT], and stable estimated glomerular filtration rate [eGFR]). Inhibition of the sympathetic nervous system (SNS) is an important and under-studied mechanism of SGLT2 inhibitors.

This systematic review explores that SGLT2 inhibitors decrease the time to first cardiovascular event or death, less heart failure hospitalizations (HFH), and reduced MACE. Improvements in renal function preserved eGFR and reduction in RRT. Also, this drug inhibits SNS further by aiding in cardiorenal protection.

## Introduction and background

Data from the United States shows that diabetes mellitus (DM) affects roughly 11.3% of the population. It causes significant microvascular and macrovascular complications. Significant microvascular alterations cause chronic kidney disease (CKD), and it affects 43.2% of type 2 diabetic mellitus (T2DM) patients. Cardiovascular disease (CVD) is a severe complication that affects 32.2% of DM type II patients and causes mortality in 50.3% of those who die from it [1]. Data from large-scale randomized controlled trials (RCTs) using SGLT2 inhibitors have shown considerable cardiovascular and renal improvement across various subgroups in heart failure (HF) patients. We have studied the effects of sodium-glucose cotransporter 2 (SGLT2) inhibitors in the HF population in three randomized controlled trials. The Dapagliflozin and Prevention of Adverse-Outcomes in Heart Failure (DAPA-HF) and Empagliflozin outcome trial in Patients with Chronic HF With Reduced Ejection Fraction (EMPEROR-Reduced) trials looked at the favorable effects of dapagliflozin and empagliflozin in patients with HF with ejection fraction (EF)<40, respectively [2].

SGLT2 inhibitors cardiorenal outcomes

**Figure 1:**
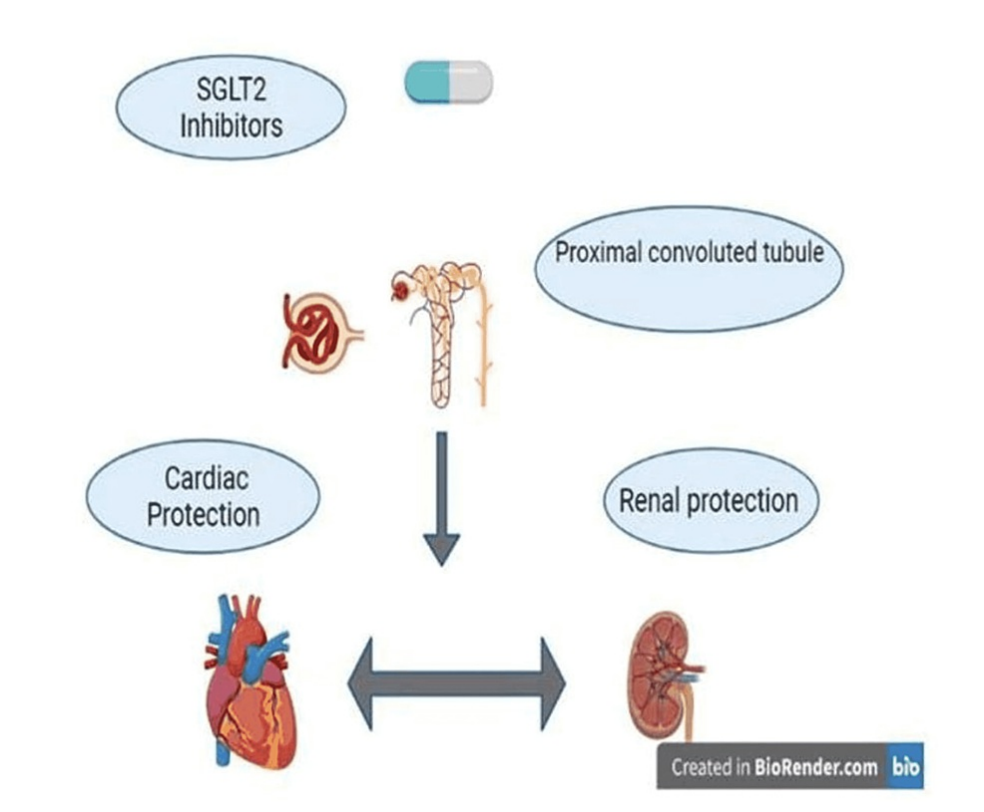
Cardiorenal effects of SGLT2 inhibitors SGLT2 - sodium-glucose cotransporter 2

SGLT2 inhibitors inhibit sodium-glucose transporter in the renal proximal convoluted tubule (PCT), with resultant loss of urinary glucose, thereby lowering serum glucose levels [3]. The data from the Empagliflozin Cardiovascular Outcome Event Trial in T2DM Patients (EMPA-REG OUTCOME) trial, which includes patients with type 2DM and CVD, there is a link between a reduction in glycosylated hemoglobin (HbA1c) and improvement in cardiovascular (CV). This relationship explains that the CV effects of empagliflozin may not be dependent on its glucose-lowering effect [4]. Sodium-glucose cotransporter 2 inhibitor (SGLT2i) reduced a composite of deteriorating eGFR, end-stage renal disease (ESRD), or renal mortality by roughly 33%, according to different meta-analyses of the data from the above-mentioned cardiovascular outcome trials (CVOTs) and other trials as shown in Figure 1 [5].

SGLT2 inhibitors pleiotropic mechanisms

**Figure 2:**
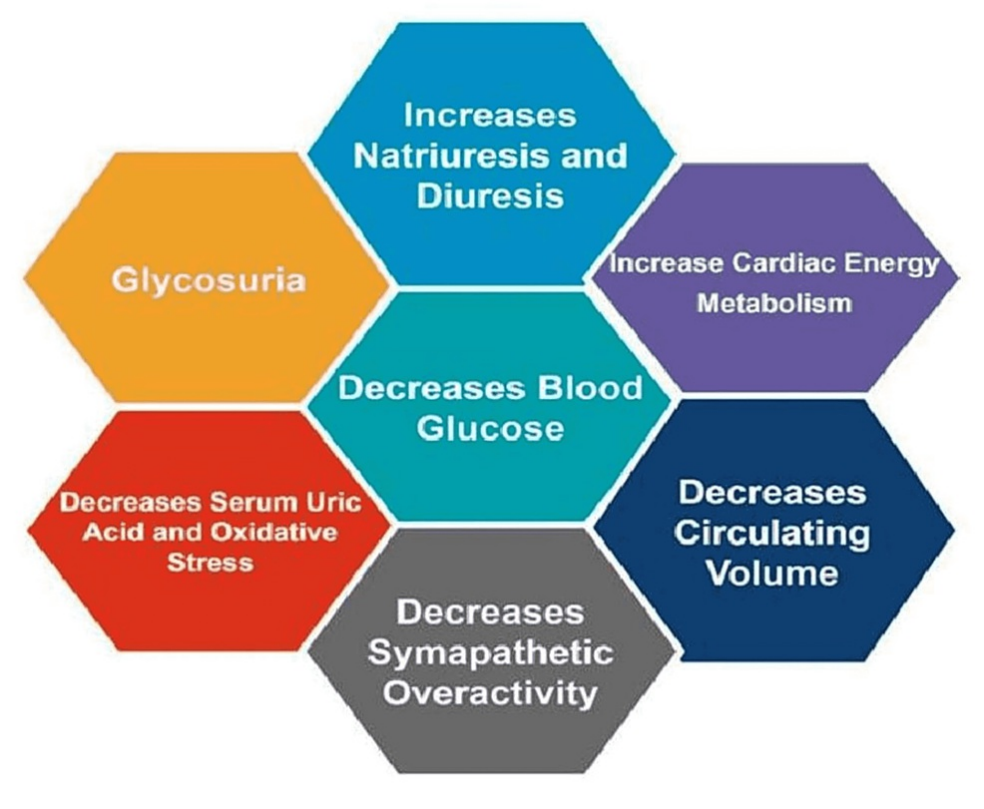
Mechanisms of action of SGLT2 inhibitors SGLT2 - sodium-glucose cotransporter 2

Even for those with CKD stage 3b, despite a minimal reduction in HbA1c, empagliflozin reduced daytime and overnight systolic blood pressure (BP). These findings distinguish antihypertensive and antihyperglycemic actions. SGLT2is can modestly decrease blood pressure. The reduction in blood pressure is thought to be insufficient to explain all the observed benefits [6]. It has been proposed that SGLT2i alters fuel consumption by increasing fatty acid oxidation and ketogenesis while simultaneously reducing carbohydrate usage [7]. When compared to healthy people, diabetics have increased blood pressure, a rapid heart rate, raised peripheral arterial resistance, and a tendency to retain sodium and fluid [8]. Through lowering intraglomerular pressure, natriuresis and altered tubular processing of filtered sodium may have substantial modulatory effects on the estimated glomerular filtration rate (eGFR), as shown in Figure 2 [9].

SGLT2 inhibitors and sympathetic nervous system

As SGLT2 inhibitors decrease blood pressure with no effect on heart rate, this led to the idea that these agents possibly attenuate sympathetic nervous activity [10]. In patients with type 2 diabetes who took optimal doses of the standard regime, including renin-angiotensin-aldosterone system inhibitors (RAAS) blockade or beta-blockers, the addition of SGLT2 inhibitors reduced heart failure hospitalization and death [8]. Similar sympatholytic effects have been observed in those with obesity (non-diabetes) [10]. Despite lower blood pressure and plasma volume, there is no increase in heart rate, which could indicate that sympathetic nervous system (SNS) activity is being slowed. SNS activity was shown to be reduced in important target organs like the heart and kidneys as more experimental and clinical data became available [11,8].

This systematic review will analyze how the SGLT2 inhibitors provide cardiorenal protection with attenuation of the sympathetic nervous system activation observed in different studies.

## Review

Method

Search Strategy

According to the reporting guidelines of Preferred Reporting Items for Systematic Reviews and Meta-Analyses (PRISMA) [12], we conducted a systematic literature review using electronic databases from January 2016 to February 16, 2022. The databases we searched included PubMed, Google Scholar, and Biomed Central. 

Medical Subject Heading (MeSH) Strategy

We used the following keywords and MeSH terms: (sodium glucose co-transporter 2 inhibitor) OR (sglt2 inhibitor)) AND (sympathetic nervous system)) OR (autonomic nervous system)) OR (sympathetic overactivity)) AND (cardiac protection)) OR (heart rate)) AND (drug effects)) AND (blood pressure)) OR (vascular stiffness)) AND (type 2 diabetes mellitus).

We also did snowball searching from the references of included articles to get the relevant data. 

Study Selection

Two reviewers independently carried out screening to recognize all potentially acceptable citations. The inclusion-exclusion criteria are shown in Table 1. We selected studies from January 2016 to February 2022. 

**Table 1:** Inclusion and exclusion criteria RCTs - randomized controlled trials

Inclusion criteria	Exclusion criteria
Studies from 2016 to 2022	Studies before 2016
Only human studies	Animal studies
Only published in English	Published in other languages
Full free texts and abstracts	Those without free access
Reviews, meta-analysis, RCTs	Non-RCTs
High-quality studies	Low-quality studies

For the final eligibility of studies, we included full-text papers that followed our inclusion-exclusion criteria.

Data Extraction

Two reviewers, Shafaat Raza (S.R.)** **and Oluwasemilore Okunlola​​​​​​​ (O.O.) extracted data independently using the standardized recording tool. The data were assessed for the type of studies and inclusion-exclusion criteria. The selected studies were searched for the content relevant to the topic as per already designed eligibility criteria. Further, the data was divided into different subheadings related to the problem being discussed. 

Methodological Quality Assessment

We used different quality appraisal tools like the Amstar checklist (for systematic reviews), the Cochrane risk of bias tool (for RCTs), and the Newcastle Ottawa scale checklist (for observational studies) to check the quality of individual studies. Each of these tools included certain criteria. Each criterion was assigned "yes", "no" or "unclear". We judged each criterion as being high, low, or moderate. If bias for every point was low or moderate, a study was regarded to be of high quality. On the contrary, if the bias for most of the domains was rated high, a study was considered of low quality. Any disagreement is resolved mutually.

Results

Literature Search

**Figure 3:**
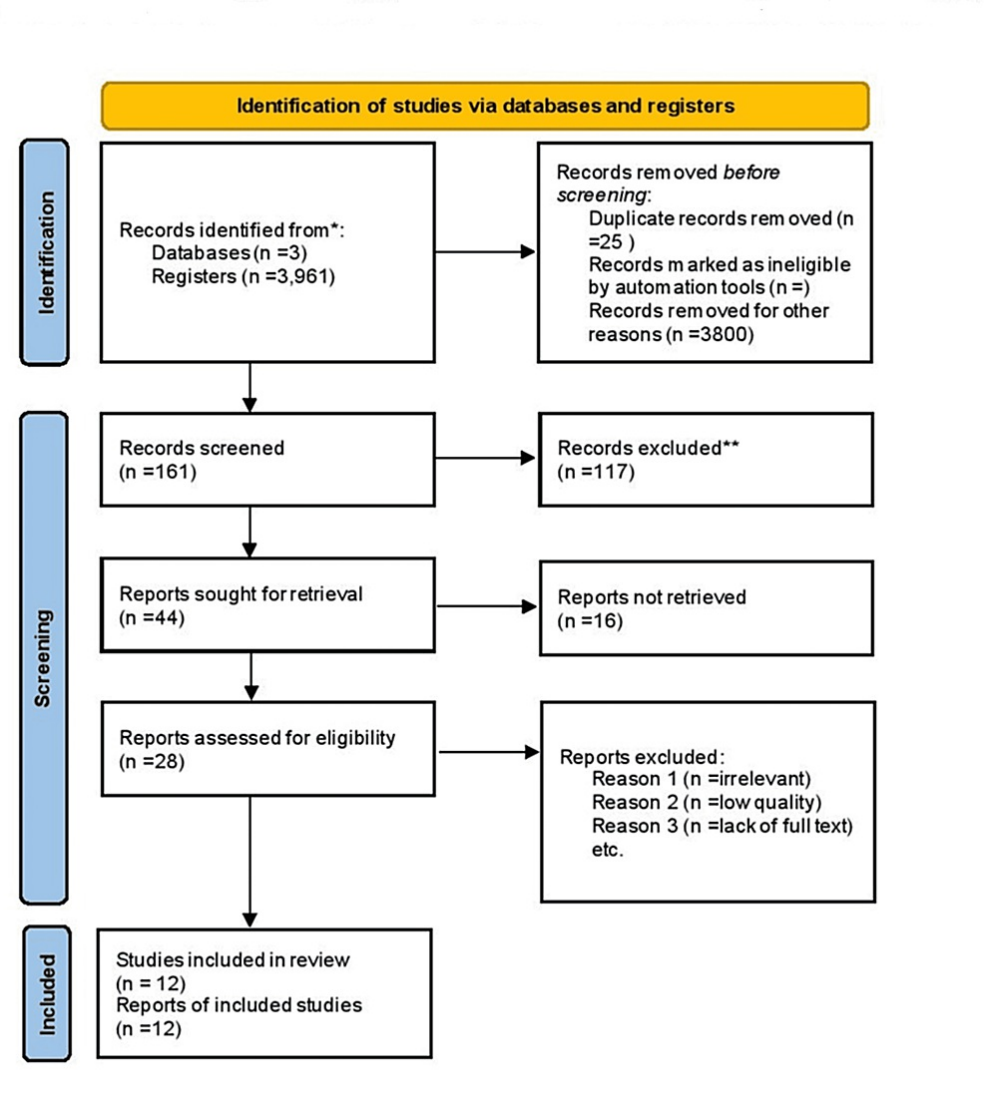
PRISMA flow diagram of included studies PRISMA - preferred reporting items for systematic reviews and meta-analyses

Figure 3 shows the flow diagram of study identification and final inclusion. Three databases PubMed, Google Scholar, and Biomed Central were searched with different search strategies and keywords. The grey literature was also searched for the data. A total of 3,961 studies were identified through all the electronic databases. Twenty-five duplicates were removed through the Zotero reference manager. Three thousand and eight hundred studies were removed after applying filters to narrow down the search. A total of 161 studies were screened. Studies that did not meet the eligibility criteria, those non-relevant to the research question, and those not available freely were removed. Six animal studies were removed. Finally, 44 studies were selected to go through the quality appraisal leading to 12 best studies being added to this review article.

Study Characteristics

The characteristic details of the studies are included in Table 2. The studies from January 2016 to February 2022 were included (Table 3). The studies included randomized controlled clinical trials, meta-analyses, and systematic reviews. Studies published in different countries like the USA, UK, Canada, Netherlands, Australia, Japan, Korea, and Taiwan were added. This review contains moderate to high-quality studies only.

**Table 2:** Study characteristics RCT - randomized control trial; CV - cardiovascular; HRV - heart rate variability; HRT - heart rate turbulence; MACE - major adverse cardiovascular events; HHF - hospitalization for heart failure; SR - systematic review; MA - meta-analyses; NOA - new-onset arrhythmias

Study	Study design	Journal	Country	No. of patients	Major outcomes
Wanner et al. [13]	Multi-centre RCT	Circulation	Germany	7020	Improved cardiorenal outcomes
Shimizu et al. [14]	RCT	Cardiovascular Diabetology	Japan	96	Reduction in HRV and HRT
Perkovic et al. [15]	Multi-centre RCT	NEJM	Australia	4401	Reduction in renal disease progression
Dagogo-Jack et al. [16]	Multi-centre RCT	BMJ	USA	8246	Improved renal outcomes
Cosentino et al. [17]	Multi-centre RCT	Circulation	Sweden	8246	Improved CV outcomes
Neuen et al. [18]	Two multi-centre RCT	Circulation	Australia	10,142	Reduction in adverse cardiorenal outcomes
Wiviott et al. [19]	Multi-centre RCT	NEJM	USA	17,160	Better CV outcomes except MACE
Petrie et al. [20]	Multi-centre RCT	JAMA	United Kingdom	4744	Reduced HHF
Toyama et al. [21]	SR/MA	Diabetes Obesity and Metabolism	Australia	7363	Reduced CV and Renal adverse outcomes
McGuire et al. [22]	MA	JAMA	USA	46,969	Reduced CV and Renal adverse outcomes
Butler et al. [23]	SR/MA	Esc Heart Failure	USA	16,820	Reduced HHF and CV events
Chen et al. [24]	Cohort study	Cardiovascular Diabetology	Taiwan	399,810	Reduced all-cause mortality and NOA

**Table 3:** Details of databases reviewed

No	Database	Total results	Results after filters	Results after eligibility and quality appraisal	Number of studies included
1	PubMed	1221	81	22	6
2	Google Scholar	5870	2571	18	5
3	Grey literature	36	8	0	0
4	Biomed Central	53	31	4	1

Risk of Bias (RoB)

Studies used in this review had a low risk of bias as assessed by quality appraisal tools. Two individuals carried out the risk of bias and quality assessment. Most of the studies included discussing either solely or in part the research question being addressed. Amstar checklist, Newcastle Ottawa scale, and Cochrane checklist were used for risk of bias assessment in individual systematic reviews, cohort studies, and RCTs, respectively. Any disagreement or conflict between the reviewers was solved with mutual understanding.

The included studies were assessed for major outcomes. Five studies showed that SGLT2i inhibits the sympathetic nervous system (SNS), which is one of the mechanisms of the major CV and renal outcomes in patients treated with these agents. These studies show that there is a relationship between the SGLT2i mechanism of action and sympatholytic benefits observed with this class of drug. Eight studies demonstrated that there is a significant reduction in cardiovascular morbidity and events, and most of these results are consistent with a reduction in sympathetic overactivity, which is considered one of the main determinants of these adverse outcomes in these patients.

Four studies elaborated on the beneficial effects of SGLT2 inhibitors on both the renal and cardiovascular systems. These studies showed a significant reduction of cardiac and renal risks and improved cardiorenal function, decreasing the threshold of pathophysiological phenomenon responsible for major cardiorenal adverse outcomes. The improvement in these systems in some parts also correlates with sympathomodulatory effects at both cardiac and renal levels with these agents. Almost all studies focused on the underlying mechanisms of the outcomes achieved with the SGLT2i, and all described the decrease in sympathetic overactivity as one of the mechanisms. Ten studies evaluated the overall effects of these agents at different levels, and results were consistent with well-observed success at many of the risk factors resulting in adverse outcomes in patients with both diabetes as well as non-diabetics.

Results from this review show that sympathetic overactivity is a major underlying mechanism for adverse cardiorenal outcomes in both diabetic as well as non-diabetic populations. These agents decrease the sympathetic nervous system activation, and this can be a very important aspect of these drugs. 

Discussion

Our review demonstrates that in patients with diabetes mellitus on the standard regime when an SGLT2 inhibitor was added to therapy, cardiovascular death, heart failure hospitalization (HFH), all-cause hospitalization, and death from any cause reduced. Our findings added to the previous evidence that SGLT2 inhibitors significantly halt the progression of renal disease and reduce its severity. Specifically, SGLT2 inhibitors significantly reduced the composite renal endpoints (progression to macro-albuminuria, serum creatinine doubling, the start of renal replacement therapy (RRT), or death resulting from the renal disease [13,14,5]. While SGLT2 inhibitors, through different mechanisms, lower blood glucose, body weight, improve hematocrit and blood pressure, as a result, predicting the net effect of SGLT2 inhibitors on CV outcomes was difficult. The processes underlying empagliflozin's renal effects are most likely complex. But direct renovascular effects could be crucial [15-17]. We came across the idea that SGLT2 participates in the inactivation of the raised sympathetic nervous system (SNS) outflow and that inhibition of SGLT2 inhibitors may have a cardiovascular protective effect through reducing renal afferent nerve activity and suppressing major mechanisms that lead to generalized sympathetic activation [15,18,19].

Cardiovascular Protection With SGLT2 Inhibitors

In the landmark EMPA-REG trial, 32% of the participants had kidney disease in addition to T2DM and preexisting CVD. When empagliflozin was added to the treatment of this high-risk population, cardiovascular death was reduced when compared to the standard of care. Heart failure hospitalizations, all-cause hospitalization, as well as all-cause mortality vs. placebo were reduced. The comparative decreases in the number of cardiac death, all-cause mortality, and HFH with empagliflozin compared to placebo were irrespective of baseline kidney function or albuminuria status [13,20,21]. Patients with heart failure with reduced ejection fraction (HFrEf), both with and without T2DM, were enrolled in the DAPA-HF trial. Dapagliflozin improved the symptoms while lowering the chance of heart failure episodes and death. Dapagliflozin decreased glycated hemoglobin in type 2 diabetes patients but had no impact in non-diabetic patients [16,18,22].

SGLT2 Classes and CV Outcomes

Although CV outcome trials using SGLT2 inhibitors in T2DM patients have yielded mixed outcomes in terms of CV death [17,23]. The observed benefits of SGLT2 inhibitors on MACE are minimal, and it has only been established in trials for canagliflozin and empagliflozin. Similarly, only empagliflozin has shown meaningful results in lowering the risk of cardiovascular death, with moderate variation among the class. The most notable CV outcome of the drug is a reduction in HHF, which is remarkably consistent throughout the class and achieves statistical significance in each trial [23].

**Figure 4:**
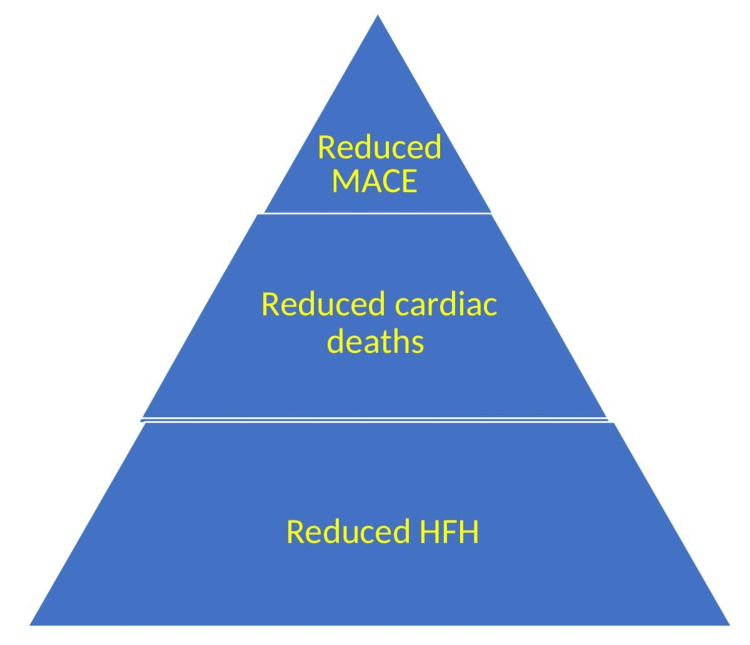
Cardiac outcomes of SGLT2 inhibitors SGLT2 - sodium-glucose cotransporter 2; MACE - major adverse cardiovascular events; HFH - heart failure hospitalizations

This is particularly important to remember the variations between the three HF trials. Severely ill patients with greater pro-B-type natriuretic peptide (Pro-BNP) levels and a poorer estimated glomerular filtration rate were enrolled in the EMPEROR-Reduced study. Patients both with and without T2DM and HFrEf were included in the DAPA-HF trial. Patients with type 2 DM with HF, regardless of ejection fraction, who were hospitalized for worsening heart failure were randomized to either sotagliflozin or placebo in the Effects of Sotagliflozin Worsening Heart Failure​​​​​​​ (SOLOIST-WHF) trial. Because it also inhibits SGLT1, sotagliflozin is different from other SGLT2 inhibitors. The exceptional consistency of all outcomes analyzed across the three trials is astounding, as shown in Figure 4.

SGLT2 Inhibitors and Renal outcomes

Previously, SGLT2 inhibitors were contraindicated for use in those with reduced eGFR when they were first approved. It was based solely on the reduction of glycemic effect as eGFR waned, and no safety profile available then that usually support eGFR-based prescriptions of the drugs. Liberalization of these limits is now justified, given the evidence of cardiovascular and renal benefits throughout the range of kidney function in patients participating in studies to date, with an eGFR as low as 30 mL/min/1.73 m^2 ^in many trials [16,22,24]. One of the distinguishing features of these agents is that their effect on urinary glucose excretion reduces with the progressive decline of kidney disease, which has been seen with the majority of the members of this class and is probably due to less number of available nephrons and subsequently diminished renal glucose reabsorption capacity, according to the Canagliflozin Cardiovascular Assessment Study (CANVAS). These facts imply that cardiorenal benefits in patients with CKD are less likely to be achieved with glucose excretion alone. This is consistent with the observation that in CKD, there is less reduction in glycosylated hemoglobin and scarcity of data suggesting reduced glycemic index to be associated with the prevention of macrovascular complications. SGLT2 inhibitors rather may have direct renal effects [15,22,25].

**Figure 5:**
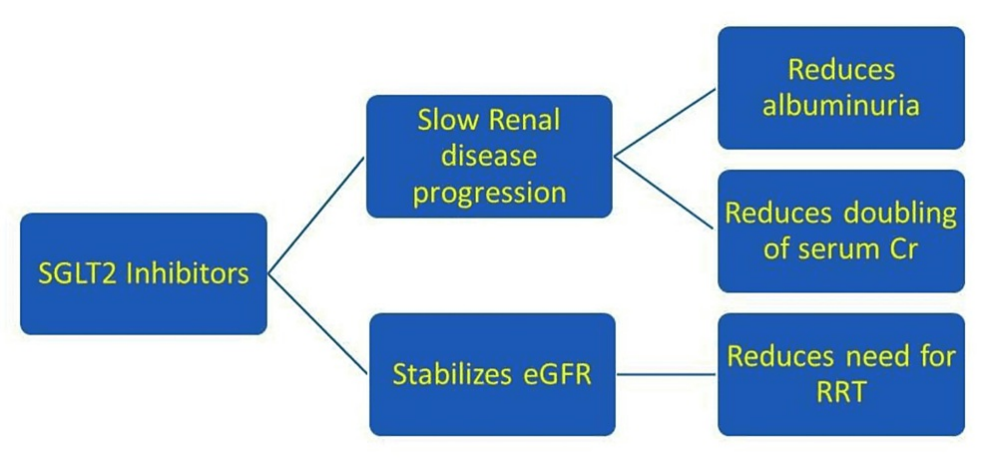
Renal protection achieved with SGLT2 inhibitors SGLT2 - sodium-glucose cotransporter 2; eGFR - estimated glomerular filtration rate; Serum Cr - creatinine; RRT - renal replacement therapy

In comparison to other glucose-lowering drugs, canagliflozin reduces the deterioration in kidney function regardless of glycemic management. The ability of these agents to increase afferent arteriolar tone by altering tubuloglomerular feedback and thus reduce intraglomerular pressure via pathways that parallel and complement those of RAAS blockade is becoming increasingly popular as a physiological explanation for their renoprotective qualities. This has been demonstrated in studies of this and other drugs in the class. With an immediate (dose-dependent) decrease in eGFR on commencement of SGLT2 inhibitors with subsequent stabilization and preservation of renal function as in Figure 5 [24,26,27].

Prespecified secondary analyses in the Evaluation of Ertugliflozin and Cardiovascular Safety trial (VERTIS CV) showed that ertugliflozin therapy delayed the degradation of kidney-filtering function when compared to placebo. Reduced albuminuria, increased eGFR (urinary albumin creatinine ratio​​​​​​​, UACR), as well as a slowed rate of advancement, and a faster rate of regression albuminuria can be found in people with a variety of renal function groupings based on eGFR baseline (CKD stage included) kidney disease, albuminuria [16,24]. Acute kidney injury AKI risk was neutral or lowered with SGLT2 inhibitors in individual CVOTs, and AKI risk was reduced by around 25% in meta-analyses incorporating cardiorenal outcome studies [18,28].

SGLT2 Inhibitors Mediated Inhibition of Sympathetic Nervous System: A New Mechanism Observed

The fact that SGLT2 inhibitors lower blood pressure without raising heart rate implies that they may be linked to a reduction in sympathetic overactivity. Both animal models of diabetes and obesity [28,29] appear to have these sympathoinhibitory effects with SGLT2 inhibitors (without diabetes). When the kidneys are harmed, the brain senses this via the afferent renal nerve, increasing sympathetic outflow from the central nervous system. Vasoconstriction of the renal vasculature, salt and water retention, and increased heart rate are all caused by sympathetic nervous system activation, and blood pressure rises as a result. A raised sympathetic tone for a long duration of time may cause advanced atherosclerosis and reduced blood flow to the kidneys, resulting in a decrease in renal function. Heart failure is also exacerbated [29]. In heart failure, overactivity of the sympathetic nervous system is linked to increased hospitalization and death. It has been demonstrated that diabetic individuals, like hypertensive patients, have an overactive systemic sympathetic tone. SGLT2 inhibitors help to reduce BP, heart rate, and edema. These hemodynamic alterations demonstrate that SGLT2 inhibitors, like renal denervation treatment, have a sympathoinhibitory impact. In other words, the mechanisms of action by which SGLT2 inhibitors act on the kidneys and diminish sympathetic overactivity could explain their preventive and therapeutic effects on HF [8,10].

SGLT2 Inhibitors, Sympathetic Drive, and New Insights

The Embody experiment was the first to examine the influence of empagliflozin (SGLT2 inhibitor) on cardiac sympathetic and parasympathetic nervous activity in patients with T2DM and acute myocardial infarction in a randomized clinical trial. It was discovered that giving an SGLT2 inhibitor to acute myocardial infarction (AMI) patients with T2DM improved cardiac nerve activity without causing any side effects [14]. Individuals on SGLT2 inhibitors (with and without diabetes) had a significant reduction in risk of all-cause death compared to non-SGLT2i users, according to population-based cohort research in Taiwan that included 399,810 patients newly diagnosed with T2M. SGLT2 inhibitor users had a 17% lower risk of new-onset cardiac arrhythmias (NOA) than non-users of the drug [23,24].

What Is the Future?

The discussion above raises some new considerations, such as whether this family of medications can be used only with the intent to reduce sympathetic overactivity instead of beta-blockers in patients with established CV diseases or in patients who cannot take beta-blockers. Another point to consider is that is, SGLT2 inhibitor is a relatively new medicine that has shown mortality benefits in heart failure patients.

Limitations and Strengths

There are a few things that this paper does well. To begin, we looked at information from systematic reviews and meta-analyses that included a substantial number of patients and were relevant to our issue. Second, we looked at the most recent randomized controlled studies that were relevant to our study, the majority of which were multi-center trials. These trials include people from many ethnic and regional groups from all over the world, and the sample sizes are large enough to make the results more general. Finally, the data is derived solely from human studies, and we have posed new research questions for future research. Our research contains certain flaws as well. We only looked at data from the last five years, so some important studies may have been overlooked. Due to possible faults in the search technique, several studies that are very relevant to our study problem may have been overlooked. Animal studies and research published in other languages are not included in this study. Studies that did not have open access or were published before 2016 were excluded.

## Conclusions

In conclusion, SGLT2 inhibitors provide cardiovascular and renal protection in type 2 diabetes mellitus patients, with suppression of sympathetic overactivity being one of the less understood but critical processes. SGLT2 inhibitors reduce cardiovascular death and hospitalization for heart failure and increase functional ability in HF patients with modest or no improvement in all-cause mortality. It also slows renal disease progression, lowers albuminuria, lowers sodium and water retention, and stabilizes the e-GFR. One of the major mechanisms for providing these benefits is SGLT2 inhibitor-mediated suppression of the sympathetic nervous system, which has received less attention in patients taking these drugs. Inhibition of sympathetic overactivity in these patients, we believe, may provide long-term efficacy and improvement amid such problems.

More research is needed to see how SGLT2 inhibitors affect the body, as this could lead to new uses for this medicine. To find this unique process and its potential ramifications, large-scale cohort, case-control, and RCT studies may be done.
